# From fragmentation to collaboration in European brain research: The early history of the European Brain Council (EBC), an organization that changed our perspective of advocacy

**DOI:** 10.1111/ene.16130

**Published:** 2023-11-13

**Authors:** Jes Olesen

**Affiliations:** ^1^ Department of Neurology, Danish Headache Center Copenhagen University Hospital Glostrup Denmark

**Keywords:** advocacy, EU research, European Brain Council, neurology, neuroscience

## Abstract

**Background:**

The European Academy of Neurology (EAN) is a member of the European Brain Council (EBC), a coalition of neurologists, psychiatrists, neurosurgeons, neuroscientists, patient organizations and industry with an interest in the brain and its diseases. It was founded by the present author. Here, its formation, early history and the results of its advocacy are described.

**Method:**

Eyewitness report and relevant literature were considered.

**Results:**

After a long and difficult inception, the European Brain Council (EBC) brought all major players with an interest in the brain and its diseases to work closely together. Important data on the cost of brain diseases, lack of funding and fantastic research possibilities were generated and effectively used in advocacy. During the early years of the collaborative effort, the funding of brain research increased from €85 million in framework program (FP) 5 to €260 million in FP6 and to more than €2000 million in FP7.

**Conclusion:**

The EBC has been extremely successful. It is essential that advocacy in the European Union continues to be united so that those involved in brain research are able to speak with one voice to policy makers. An even bigger task, still insufficiently pursued, is for national brain councils to achieve prioritization of brain research in their national political agenda to bring about improved provision of care to those living with a brain disease.

## INTRODUCTION

The roots of the European Brain Council (EBC) go back to the 1990s. There had been several attempts led independently by single European scientific organizations, such as the Federation of European Neuroscience Societies to improve the funding of neuroscience at the European Union (EU) level but to no avail. Funding for neuroscience continued to be much lower than funding for cardiovascular diseases or cancer. Even the last attempt to increase funding led by eminent basic scientists was not successful. The present author, Jes Olesen, had paid close attention to these attempts, but had been busy developing the European Federation of Neurological Societies (EFNS) during the early 1990s. After the above‐mentioned meetings he did, however, have time and energy to think about the overarching problem of EU funding of brain research.

The striking new idea for success was to move from man to molecule and not from molecule to man. From bed to bench and not from bench to bed. It was equally clear that much competition and mutual mistrust existed between the players interested in the brain and its diseases. The Commission for Research did not want to talk to several different organizations who were independently lobbying for funds for their respective fields. Finally, and most importantly, it became apparent that patient organizations were crucial partners in the bid to have a real impact on the political scene. For a few years these thoughts were tested in discussions with many stakeholders. It became clear that we needed to attract the attention of policy makers and of the general public. However, this was not an easy endeavor. First, we needed support from neurological patients themselves, but there was no suitable organization at the European level.

## THE FORMATION OF THE EUROPEAN FEDERATION OF NEUROLOGICAL ASSOCIATIONS

Jes Olesen met with Mrs Mary Baker, President of the Parkinson Disease Association in the United Kingdom and consultant at the World Health Organization (WHO) in the late nineties. She immediately understood the importance of uniting all players in the field of brain disease and brain research and was willing to spearhead the formation of a European Federation of Patient Associations (EFNA). It was possible for Jes Olesen, as President of the EFNS, to start a close collaboration between the EFNS and the European Parkinson's Disease Association including establishing a permanent office led by Mrs Eveline Sipido. A first problem to overcome was that European patient associations were national and only few European federations existed, such as the European Parkinson's Disease Association. Therefore, the task was first to stimulate national patient associations for each disease to form a European federation and then to ask these federations to form the overarching association of all neurological patient associations. Mary Baker and Eveline Sipido did most of this work but Mr Alistair Newton, President of the European Dystonia Association was a great help. He continued later with invaluable work as treasurer of the EBC. In 2000, the EFNA was formally founded, with Mary Baker as President. Indeed, this represented a milestone for European collaboration.

## FINDING PARTNERS FOR THE EBC

The EFNS and EFNA together represented the seed for the development of an umbrella organization which could merge all major players in the area of brain research, and it was possible to start discussing how to enroll the other major stakeholders. At that time there were two main European psychiatric organizations: the newly born European Psychiatric Association (EPA) and the European College of Neuropsychopharmacology (ECNP) representing biological psychiatry. The latter was well established and already collaborated with neurology and neuroscience organizations. ECNP immediately agreed to participate while the EPA was admitted later. It was a bigger challenge to involve psychiatric patients. The epoch of anti‐psychiatry and “drug‐free psychiatry” was not yet over. We identified the Global Alliance of Mental Illness Networks (GAMIAN), led by Mr Rodney Elgie. This organization was primarily concerned with depression and bipolar disorder. Rodney Elgie immediately agreed to be a partner. There was only one organization for neurosurgery, the European Association of Neurosurgical Societies (EANS), and it quickly joined. From health insurance we had support from Mr Peter Buschmann of the German *Allgemeine Ortskrankenkasse* (AOK), but there was no European organization of insurers available. We decided that we would include only one organization to represent each of the major subfields of brain diseases or brain research in the core committee. On the one hand, by keeping the number of voting members low we hoped to optimize efficiency of operation. On the other hand, we aimed for broad and inclusive participation, and therefore we decided to admit all other members as observers. Soon the EPA was also made a voting member because the field of psychiatry was so vast and, later, the policy was made even more liberal. In fact, it proved relatively easy to reach agreement and almost all decisions were made unanimously.

Another important stakeholder was the large pharmaceutical industry. Iits European association, the European Federation of Pharmaceutical Industries and Associations (EFPIA).

Did not want to participate to avoid a conflict between different health‐related areas, such as cardiovascular and cancer, which might have felt excluded. Instead, a win‐win strategy was found which was to form a board of pharmaceutical companies with the right to elect one voting member to the EBC board. Finally, also included, and in fact completely indispensable, were the basic neuroscientists in the Federation of European Neuroscience Societies (FENS). The President of this body at first hesitated and expressed his skepticism. Nonetheless, other members of the Executive Committee of FENS had a clear vision of the importance of the EBC to advocate for neuroscience. Guided by their Secretary General Monica DiLuca, they worked hard to join and FENS became a voting member. Furthermore, and very importantly, the new organization was strongly supported by the Commission for Research, not least director Dr Octavi Quintana Trias, who recommended that all players should join forces in the EBC.

The EBC became a privileged interlocutor, bringing the needs of the brain research community as a whole to the European Commission and to policy makers in the European Parliament.

## SCOPE AND AIMS OF THE EBC

**FIGURE 1:**
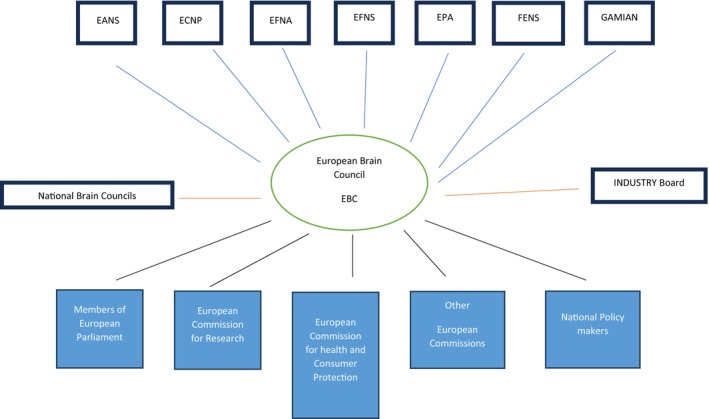
Organogram of the European Brain Council (EBC). We took care to show that the EBC was under the member organizations, not above, as at first there was widespread fear that the EBC could in some way control the member organizations, when in fact it was their servant. EANS, European Association of Neurosurgical Societies; ECNP, European College of Neuropsycho Pharmacology; EFNA, European Federation of Neurological Associations; EFNS, European Federation of Neurological Societies; EPA, European Psychiatry Association; FENS, Federation of European Neuroscience Societies; GAMIAN, Global Alliance of Mental Illness Networks.

From the beginning there was much concern about this new organization, the structure of which is shown in Figure 1; the member organizations were afraid to be overruled or controlled by other members or by the EBC itself. It was extremely important to clarify from the beginning that the EBC would never embrace activities that were naturally covered by its member societies. The main activity of the EBC was, and still is, to advocate for the brain and its disorders in general. It was a long haul to create trust. With the wisdom of hindsight, we probably chose the wrong name; it should have been the European Brain Coalition and not the European Brain Council. We made a great effort to demonstrate that the EBC would never overrule any member organization and defined the working remit of the EBC to include only questions of an overarching nature involving at least two of the member organizations and preferably more. We also emphasized that the EBC should normally work by consensus and this has almost always proven to be the case [1]. There was also the age‐old envy when it came to allocation of research money to basic and clinical science. We strongly emphasized that the aim of the EBC was to increase the size of the pie and that any discussion of cutting it up should be deferred until the pie size had been decided by the European Commission. The motto was: let us prefer a modest slice of a big pie to a bigger slice of a small pie. Lastly, there was the question of nomenclature. The term “neuroscience” sounds good to a scientist, but it does not sound so good to politicians, decision makers and the general public. It also does not sound good to psychiatrists and only partly good to neurologists. In parallel to having the name “European Brain Council”, we decided to talk about brain research and brain diseases but emphasized that this does of course include the spinal cord, peripheral nerves and muscle. It was proven later to be a wise decision because the names brain diseases and brain research are easy for everybody to understand, and compete well with cardiovascular diseases and cancer.

## ADVOCATING FOR BRAIN RESEARCH

**FIGURE 2:**
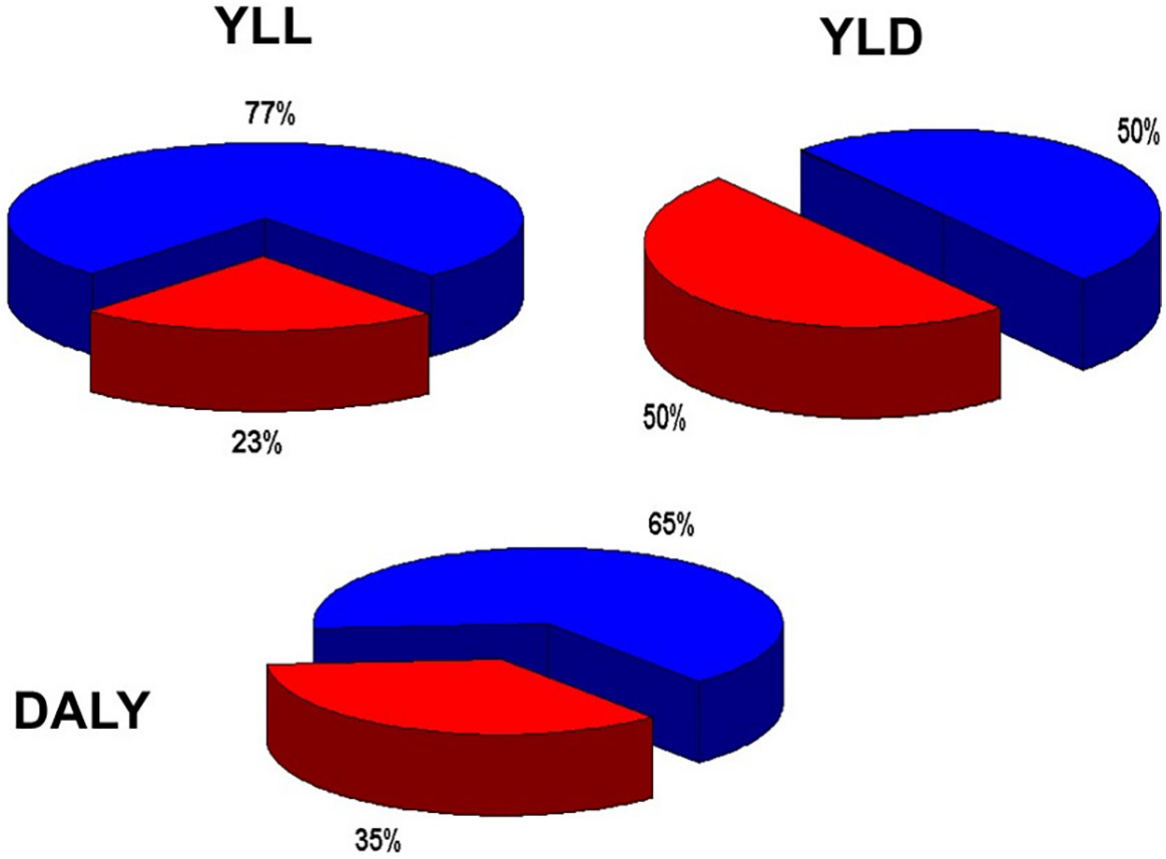
World Health Organization data on the burden of brain diseases were provided in different places: brain tumors under cancer, neuroinfections under infections, etc. When data from all the different types of brain disease were gathered, the burden of brain diseases was an astounding 35% of the burden of all diseases [2]. These data were very beneficial in the drive for more funding to brain research. DALY, disability‐adjusted life‐years; YLD, years lost to disability; YLL, years of life lost.

Time was short if we were to have an influence on the so‐called sixth framework program (FP6) for research. This was to take place in the years 2003–2006 and the EBC was only officially incorporated in 2002. Already in 2001, we met with the commissioner for research, Philippe Busquin, about the draft proposal for FP6. There was no mention of brain diseases or of neuroscience in that draft. We presented the WHO with data collated by Matilde Leonardi and Jes Olesen, demonstrating that brain diseases accounted for 35% of all disease burden (Figure 2). This, and the fact that we spoke on behalf of all disciplines (although not yet incorporated) with an interest in brain diseases and brain research, had a great impact. During the continued interaction with the Commission, we achieved recognition of brain diseases in the major disease program as just one out of four groups of diseases. Just a few words in the final program but worth millions of Euros. More specifically, support increased from 85 million Euros in FP5 to 260 million in FP6.

Nevertheless, there was no time to rest. We had been extremely lucky to get through the door at the very last moment for FP6 and now the Commission had already started preparing FP7. A good starting point was a conference held by the Commission for Research in September 2003, at which all players were present, and where the EBC played a big role. It was the final endorsement of the EBC as the single representative for brain diseases and brain research in Brussels. In parallel, the EBC had developed its economy, its constitution and bylaws, and had obtained its own headquarters at the *Club Universitaire* in Brussels. From there, Mrs Eveline Sipido and the Executive Directors that followed at too short intervals, could continuously be in contact with the European Commission. Together with the patient organizations EFNA and GAMIAN and, in some cases, the disease‐specific patient organizations, a series of lunch meetings were organized for members of the European Parliament, and we also had the opportunity to speak at the European Parliament.

## EVIDENCE AND HOW TO USE IT

**FIGURE 3:**
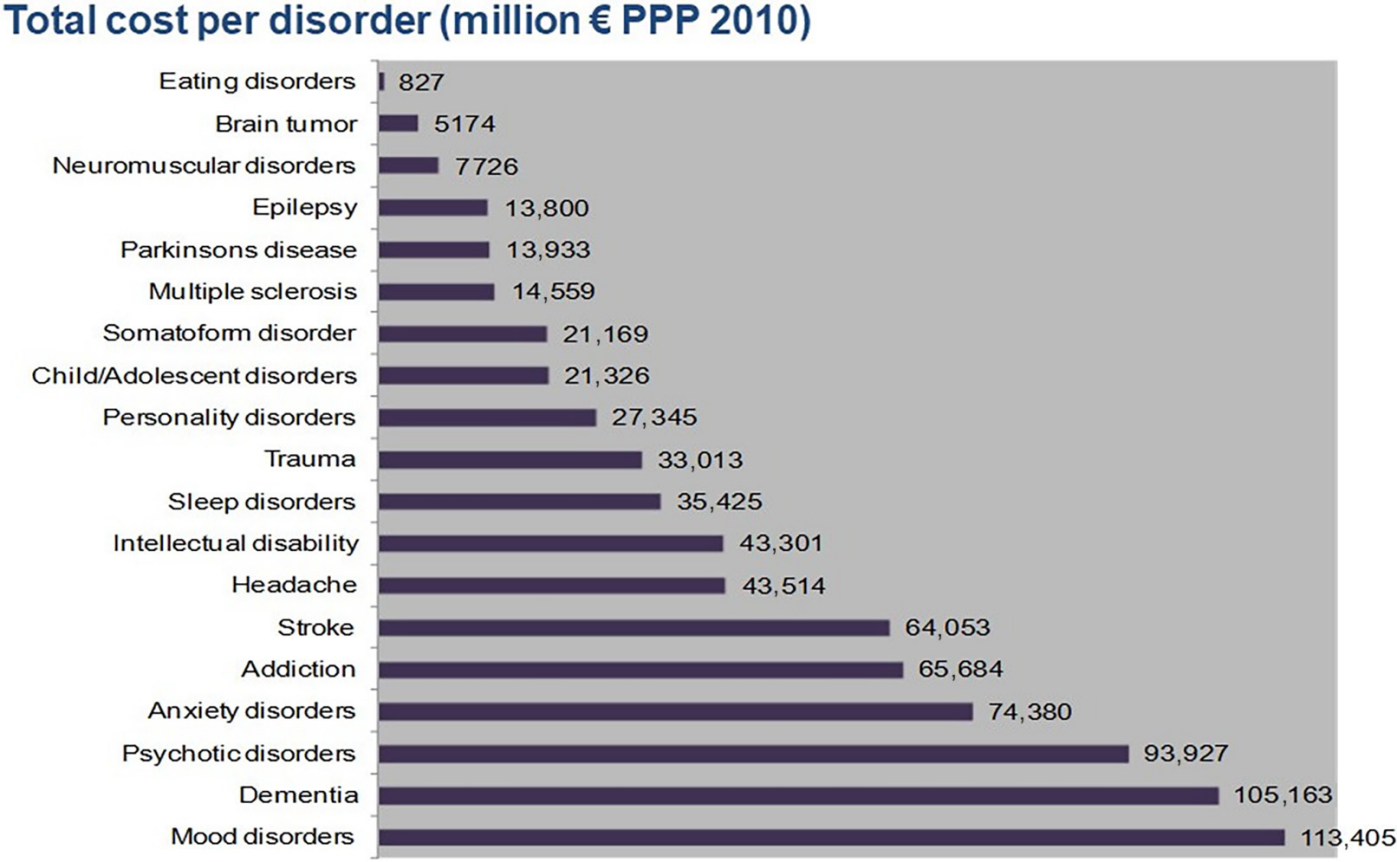
Total cost per disorder (million Euros PPP 2010). The cost of disorders of the brain in 2010 was 798 billion per year in this second study [4]. The first study estimated a cost of 386 billion which was enough to greatly impact the framework program 7, which ended with 2000 million Euros for brain research.

Advocacy in Brussels is everything. If you are not there, then others replace you and there is continuous pressure on resources. However, advocacy without data is generally useless. The EBC had to generate a lot of strong data in addition to the paper by Olesen and Leonardi [2]. Together with Professor Bengt Jönsson, Stockholm Health Economics—later European Health Economics—and Professor Hans‐Ulrich Wittchen, we conducted a huge study, “Cost of disorders of the brain in Europe”, which was published in 2005 [3]. It included estimates of the costs of major brain diseases (neurological, neurosurgical and psychiatric) and concluded that the total cost was 386 billion Euros per year in Europe. This figure exceeded the cost associated with cancer or cardiovascular diseases. Data from a later study [4] showing even greater cost are shown in Figure 3.

**FIGURE 4:**
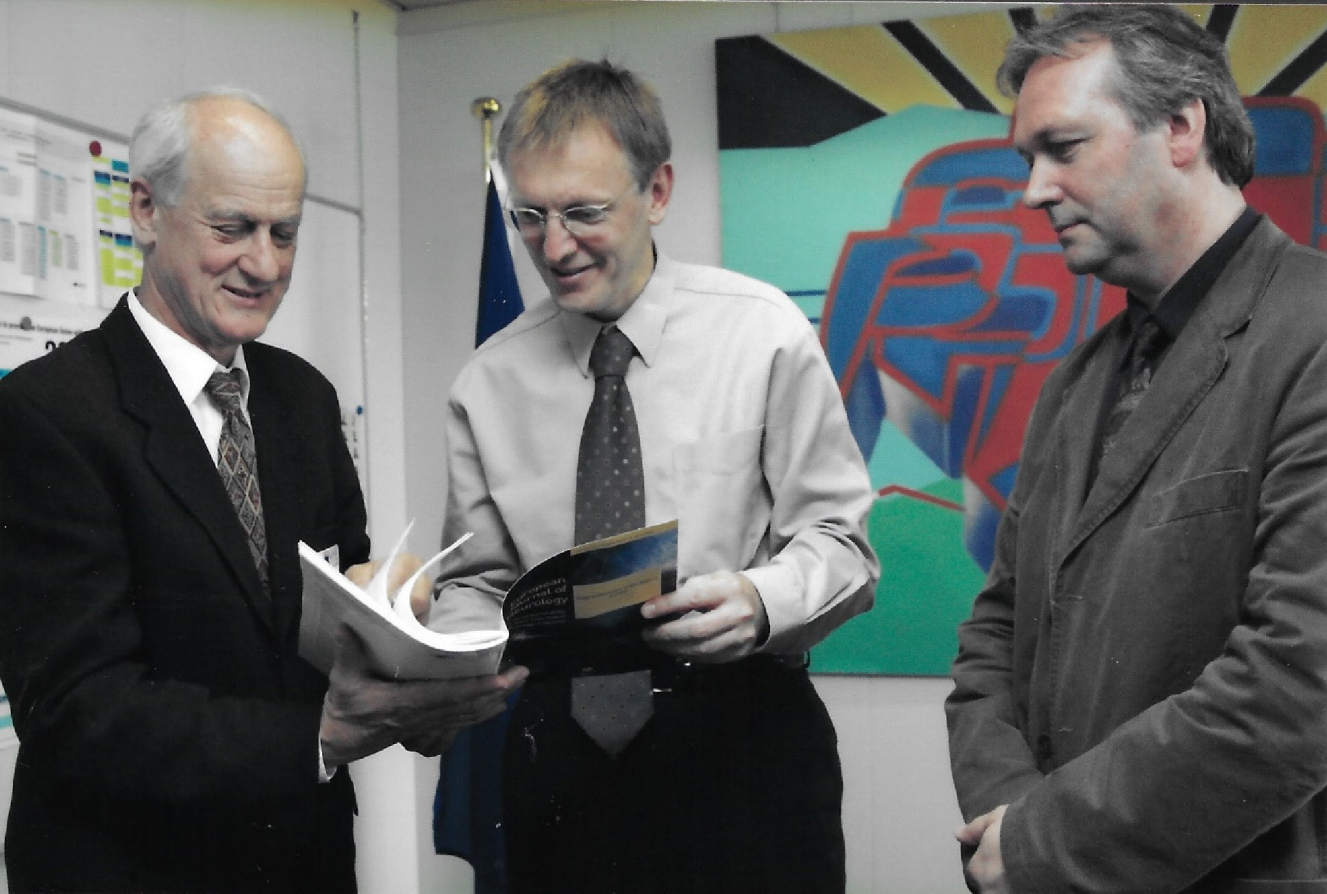
Professor Tamas Freund president of the Federation of European Neuroscience Societies and Jes Olesen President of the European Brain Council presenting the “Cost of disorders of the brain” publication to Commissioner for Research Janez Potocnik.

The “Cost of disorders of the brain” publication was presented to Commissioner for Research Janez Potocnik. He immediately accepted the importance of brain diseases. It was an immense success: we simply needed a change in perspectives on brain research in Europe. We needed to improve the lives of our fellow Europeans living with these life‐impeding disorders. We needed adequate support for robust research, improved care and a society that prioritizes the health and wellbeing of all its citizens Figure 4.

Together with Professor Jönsson, we also conducted a study of resource allocation to brain research in Europe [5]. The study showed there was a very modest allocation of resources considering the high cost and burden of brain diseases. Therefore, arguments were very strong that funding for brain research should be considerably increased in FP7. To further pave the road, we developed a so‐called Consensus Document on European Brain Research [6], which showed that we have a strong base for this kind of research in Europe, and that further investment in brain research would have tangible benefits for Europe's population. This document was also meant to help the Commission in deciding calls for FP7. This happened later when topics from the Consensus Document were used for several calls. Both the resource paper and the consensus paper were sent in drafts at the earliest possible time to the European Commission. Both these papers were instrumental in helping policy makers define priorities in the area of brain research.

## WAS EBC ADVOCACY EFFECTIVE AT THE EU?

As the FP7 unfolded we were surprised to see the figures for support of brain research. There are many different programs involving the brain and our initial focus was on the major disease area. The figures looked good, but were eclipsed by data from the Commission itself. A next‐to‐final estimate was that brain research had received 1.4 billion Euros. The last estimate after conclusion of the program said, however, that when all aspects were taken together, we reached an astounding 2 billion. This was 20 times the amount awarded under FP6 and 60 times that awarded under FP5. The advocacy of the EBC thus proved extremely successful at the EU level. Would this have happened without the EBC? It seems highly unlikely. Both the cardiovascular field and cancer are strongly represented in Brussels and continuously lobby the Commission. In the absence of the EBC there would be little defense for brain diseases and brain research. The rules of the game are such that absence of advocacy means that you lose.

The activities of the EBC became more and more incisive in the following years. As an example of success, the EBC led the European Brain Research Area (EBRA) project, which was funded by H2020 and created as a catalyzing initiative for brain research stakeholders (researchers, clinicians, patients, governments, funders and public institutions) to streamline and better coordinate brain research across Europe while fostering global initiatives. The Consortium of this EU‐funded project consisted of the EBC, the Network of European funding for Neuroscience research (NEURON), Joint Programme – Neurodegenerative Disease Research (JPND) and the Human Brain Project (HBP). Through the EBRA project, key advancements have been made in developing a shared strategic research and innovation agenda—the Shared European Brain Research Agenda (SEBRA) —currently being discussed among key players in the brain space, with the aim of providing recommendations on future areas for excellent, innovative, and translational research.

## NATIONAL ADVOCACY

To really change the funding situation for brain research, it was necessary to consider the national level, as 90% of research funding and 100% of healthcare is provided at this level. How could we use our enormous success at the EU to have an impact at the national level? We had hard data from the study on cost of disorders of the brain study for every European country. Jes Olesen wrote a paper entitled “Cost of disorders of the brain in Denmark” [7]. We then sent the Danish paper and data for each country to selected national representatives, asking them to write national papers and inviting them to copy without hesitation the Danish draft manuscript (this was before the frequently annoying computer programs evaluating plagiarism) and several countries did that [8]. We realized that it was not only the cost data but the whole EBC concept of collaboration that needed to be copied at the national level. Hence, we charged national leaders to form alliances of all players at the national level as the EBC had done. The Belgian Brain Council was the first, founded under the leadership of Professor Jean Schoenen, and soon others followed. Unfortunately, this development has not yet been completed. It is indeed very difficult for all players in a country to unite as questions arise such as “Are others going to take my cake?” or “Will the professionals cheat the poor patients?”. When a formal and financially independent brain council could not be formed, we recommended a more informal action group without incorporation. This could interact with the EBC and advocate for brain research and brain diseases. Eventually it could develop into a national brain council. At present the EBC collaborates with several national brain councils but much more is needed at the national level.

## THE FUTURE OF COLLABORATION AND ADVOCACY

Such was the birth and early life of the EBC, but it continued to be very active in its advocacy. A second version of the cost document showed that costs were in fact 798 billion [4]. Monica Di Luca also developed an updated version of the Consensus Document on Brain Research in Europe [9] and there have been many other activities that would best be described by one of my successors. Looking at this early history, it is almost a miracle that it was possible to create the EBC and that it achieved its aims of increasing the focus on brain research and brain diseases. Looking to the future, there are, however, worries and unmet needs. Broad collaboration with regard to the brain is necessary but neurology, psychiatry and basic neuroscience now do much more on their own in Brussels. This can be a strength, but is more likely to weaken the EBC and we run the risk of “losing our edge”. The enormous success of the EBC in the EU has not been repeated sufficiently at the national level. National European governments still do not prioritize brain diseases and brain research. In Denmark, despite the experience and efforts of Jes Olesen, collaboration stalled because patient organizations feared dominance by doctors and feared that their resources could be used in ways outside of their control. Nothing could eliminate this paranoid attitude. Others may meet it in other countries, so we warn colleagues to be prepared.

## CONCLUSIONS

The brain research community demonstrated through the EBC a clear need: the European Commission must come forward with a clear plan to tackle brain health in a collaborative, integrated and forward‐looking manner in Europe. Additionally, Member States and associated countries would benefit from the implementation and creation of brain research programs addressing brain health in a systematic and comprehensive manner.

As the Horizon Europe framework program is fully underway, the momentum can only be accelerated through an ambitious partnership in brain health in Europe. It can develop a common goal with prioritized topics and ensure that brain research is recognized as essential for society. A European Brain Health Partnership involving member states is now fundamental to improve alignment and synergies across European initiatives and to intensify scientific collaborations, identify gaps in knowledge and improve data sharing. It is necessary to move toward a future where citizens and society will benefit maximally from the breakthroughs in brain research.

We are all looking forward to this new challenge.

## AUTHOR CONTRIBUTIONS

**Jes Olesen:** Writing – original draft.

## CONFLICT OF INTEREST STATEMENT

None.

## Data Availability

Data sharing not applicable to this article as no datasets were generated or analysed during the current study.
